# Effects of Loading Frequency and Loading Type on High-Cycle and Very-High-Cycle Fatigue of a High-Strength Steel

**DOI:** 10.3390/ma11081456

**Published:** 2018-08-16

**Authors:** Yuanpei Hu, Chengqi Sun, Jijia Xie, Youshi Hong

**Affiliations:** 1 LNM, Institute of Mechanics, Chinese Academy of Sciences, Beijing 100190, China; huyuanpei@lnm.imech.ac.cn (Y.H.); scq@lnm.imech.ac.cn (C.S.); xiejj@lnm.imech.ac.cn (J.X.); 2 School of Engineering Science, University of Chinese Academy of Sciences, Beijing 100049, China

**Keywords:** loading frequency, loading type, very-high-cycle fatigue, fatigue strength, high-strength steel

## Abstract

High-cycle and very-high-cycle fatigue tests via rotary bending (52.5 Hz), electromagnetic resonance (120 Hz) axial cycling, and ultrasonic (20 kHz) axial cycling were performed for a high-strength steel with three heat treatment conditions, and the effects of loading frequency and loading type on fatigue strength and fatigue life were investigated. The results revealed that the loading frequency effect is caused by the combined response of strain rate increase and induced temperature rise. A parameter *η* was proposed to judge the occurrence of loading frequency effect, and the calculated results were in agreement with the experimental data. In addition, a statistical method based on the control volume was used to reconcile the effect of loading type, and the predicted data were consistent with the experimental results.

## 1. Introduction

The research of very-high-cycle fatigue (VHCF), which is fatigue failure beyond 10^7^ cycles of loading, is a hot topic in structural integrity because engineering structures require greater than 10^7^ cycles of safe performance [1,2,3,4,5,6]. The major feature of VHCF is its loading stress lower than the traditional fatigue limit and fatigue life longer than that of high-cycle fatigue (HCF) [7,8]. Crack initiation of VHCF is mostly from the interior of a specimen with the morphology of a fish eye (FiE) containing a fine-granular area (FGA) [9] surrounding an inclusion as crack origin, and more than 95% of total fatigue life is consumed in FGA regions [10]. The influencing variables on internal fatigue crack initiation include stress level, microstructure, inclusion size, etc., and the effect of stress concentration factor and corrosive environment on VHCF behavior was also investigated [11]. Due to the fact that the FGA region consumes a dominant part of total fatigue life, the formation mechanism of FGA has been extensively investigated [3,12,13,14,15,16,17,18,19], and the recently proposed model of numerous cyclic pressing [18,19] is capable of explaining the formation mechanism of FGA regions for the cases subjected to different stress ratios, which is also validated by new results on titanium alloys [20].

Several experimental devices have been used for the testing of VHCF, such as rotary bending (RB), servo-hydraulic (SH), electromagnetic resonance (ER), and ultrasonic loading (UL) machines. For the reason that the conventional loading (CL) frequency of RB, SH, and ER machines cannot satisfy efficiency in terms of experimental time in VHCF testing, UL machines have been widely used thanks to their ultra-high loading frequency, e.g., 20 kHz, but the increase in loading frequency will influence the fatigue strength and fatigue life in the VHCF regime, for which quite a number of investigation results [21,22,23,24,25,26,27,28,29,30,31,32,33,34] have been reported. Furuya et al. [22] performed fatigue tests at the frequencies of 100 Hz, 600 Hz, and 20 kHz for a low-temperature-tempered steel with the results showing that loading frequency had little effect on VHCF properties. Zhu et al. [24,25] studied the fatigue stress–life (S-N) behavior of an aluminum alloy at the frequencies of 75 Hz and 20 kHz, and reported that the fatigue life in air at 20 kHz was 5 to 10 times longer than that at 75 Hz at the test temperatures (20 °C, 150 °C, and 250 °C). Li et al. [27] investigated the VHCF properties at the frequencies of 95 Hz and 20 kHz for a high-strength steel, and showed that the values of fatigue strength tested with both frequencies were within the same scatter band. Guennec et al. [28] carried out fatigue tests at frequencies of 0.2 Hz, 2 Hz, 20 Hz, 140 Hz, and 20 kHz for a structural steel, and showed that the fatigue strength increased with the applied loading frequency. Zhao et al. [34] investigated the effect of loading frequency at 52.5 Hz and 20 kHz for a high-strength steel with four strength conditions, and the results indicated that the fatigue strength at 20 kHz was slightly lower than that at 52.5 Hz for the highest strength condition and was higher than that at 52.5 Hz for the conditions with relatively low tensile strength. Above all, loading frequency is certainly a factor to affect the fatigue strength of metallic materials, but it seems that for some materials this effect is evident whereas for some other materials such an effect is diminishing.

Researchers have proposed explanations to understand the effect of loading frequency. In an early overview [35] and a recent review [36], the effects of loading frequency on cyclic plastic deformation, dislocation movement, damage localization, and fatigue crack growth in fcc (face-centered cubic) and bcc (body-centered cubic) metals were addressed. For fcc metals, the activation energy for dislocation movement is relatively low, which results in a low value of critical shear stress required for plastic deformation, and the sensitivity of shear stress to strain rate is also low. Thus, the slip systems of fcc metals are active under high frequency and the frequency effect is less noticeable. To the contrary, bcc metals possess relatively large values of dislocation activation energy and critical shear stress, which results in the slip systems tending to be less active under high frequency, so the effect of loading frequency is pronounced. Papakyriacou et al. [37] also showed that for bcc and hcp (hexagonal close-packed) metals under high frequencies, the dislocations were less active and the fracture mode tended to transition from ductile to brittle. The effect of loading frequency for alloys is more complicated due to the dislocation movement strongly impeded by interstitials, second phases, and inclusions. Yet, the mechanism of loading frequency effect on fatigue performance for metallic materials is still unclear, and needs further investigation.

In addition to the effect of loading frequency, the effect of loading type also exists in fatigue experiments. The common loading types are axial loading (AL), RB, three- or four-point bending, and torsional loading, and several investigations [27,38,39,40,41,42,43] have investigated the topic of loading type effect. Murakami et al. [38] first proposed the concept of control volume to interpret the effect of loading type in terms of the control volume which was defined as the volume bearing ≥90% of the maximum loading stress. Li et al. [27], Shiozawa et al. [39], and Nakajima et al. [40] accomplished VHCF tests under conventional axial loading (CAL) and RB, and the fatigue strength under RB was higher than that under CAL. The values of control volume for the specimens under the two loading types were used to estimate the fatigue limit, in order to explain the difference in the test data between RB and CAL. Li et al. [41] also showed that the size of activated inclusion and FGA increased with the increasing control volume of the specimen. Recently, Sun et al. [43] proposed a statistical method to predict the fatigue strength under different control volumes, and the predicted results were in good agreement with experimental data. In the derivation of such a statistical method, data scattering was described by Weibull distribution, which is commonly used in the analysis of specimen size effect, e.g., a recent paper by Zhang et al. [44] addressed the effect of sample size on Weibull parameters. It is clear that for the feature of stress gradient under RB, the control volume of the specimen is substantially smaller than that under AL; thus, the AL specimen has a higher risky possibility than the RB specimen, which leads to lower fatigue strength by the AL method than by the RB method. However, research explaining the loading type effect using control volume is still limited.

The aim of this paper is to further investigate the effects of loading frequency and loading type on HCF and VHCF performance for high-strength steels. For this purpose, fatigue tests up to VHCF regime for a high-carbon chromium steel (GCr15, equivalent to SAE 52100 or SUJ2) with three heat treatment conditions were conducted under RB, electromagnetic resonance axial loading (EA), and ultrasonic axial loading with cooling (UA) and without cooling (UA-NC). During EA, UA, and UA-NC tests, the strain rate of the specimen with three heat treatment conditions was readily computed and the temperature on the specimen surface was monitored via a thermocouple system. In addition, monotonic loading tests at room temperature under three strain rates (10^4^ s^−1^, 5 × 10^−3^ s^−1^, and 500 s^−1^) and at high temperatures (100 °C and 200 °C) under 500 s^−1^ were carried out to investigate the effect of strain rate and temperature on material strength. As a consequence, the combined effect of strain rate and temperature on material strength was used to analyze the effect of loading frequency. In addition, a statistical method based on the control volume was adopted to reconcile the effect of loading type.

## 2. Material and Methods 

### 2.1. Test Material

The test material in this research is a high-carbon chromium steel (GCr15, equivalent to SAE 52100 or SUJ2) with the chemical composition (wt %) 1.00 C, 1.52 Cr, 0.31 Mn, 0.21 Si, 0.0086 P, 0.016 S, and Fe balance. The machined specimens were heated at 845 °C for 10 min in a salt-bath furnace, then oil-quenched and tempered in vacuum for 2 h at 150 °C (TT 150), 200 °C (TT 200), or 400 °C (TT 400). The microstructure for TT 150 mainly contains tempered martensite, carbides, and retained austenite, and the microstructure for TT 200 mainly contains tempered martensite and carbides, while the microstructure for TT 400 contains tempered troostite and carbides. The tensile strength under strain rate 10^−4^ s^−1^ and the microhardness for the three conditions are listed in Table 1. The strength values for TT 150 and TT 200 are evidently higher than that for TT 400.

### 2.2. Experimental Methods

**Figure 1:**
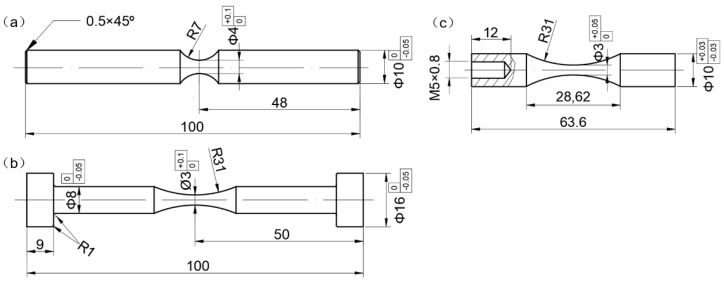
Shape and dimensions (mm) of fatigue test specimens: (**a**) rotary bending (RB) specimen; (**b**) electromagnetic resonance axial loading (EA) specimen; (**c**) ultrasonic axial loading with cooling (UA) or without cooling (UA-NC) specimen.

Fatigue tests were conducted with RB, EA, and UA testing machines (GF-20-TC, Lasur Corporation, Paris, France), and the loading frequencies for the three kinds of testing machines were 52.5 Hz, 120 Hz, and 20 kHz, respectively. All fatigue tests were performed in air, at room temperature, and with a stress ratio of *R* = −1. All the specimens are of the hourglass type, and the geometry of the specimens is shown in Figure 1. For the RB specimen, the minimum diameter is 4 mm and the round notch radius is 7 mm. For the EA and UA specimens, the gage section is the same with the minimum diameter of 3 mm and the round notch radius of 31 mm. Note that the UA specimen is specially designed so that the resonant frequency of the specimen in axial tension and compression is well matched with that (20 kHz) of the transducer of the ultrasonic testing machine.

For the purpose of investigating the effect of temperature rise of the specimen caused by ultrasonic frequency, the fatigue tests were performed with compressed air cooling (UA) and without cooling (UA-NC) on the specimen surface. A thermocouple system (made by the authors) was used to monitor the temperature on the minimum cross-sectional surface of EA, UA, and UA-NC specimens. In addition, tests with separated Hopkinson pressure bar were carried out for testing the mechanical property of the three conditions at high strain rate, and a muffle furnace was equipped for testing at high temperature.

The fracture surfaces of most failed specimens were examined by using a field-emission type scanning electron microscope (SEM, JSM-IT300, JEOL, Tokyo, Japan).

## 3. Fatigue Testing Results

### 3.1. S-N Data

**Figure 2:**
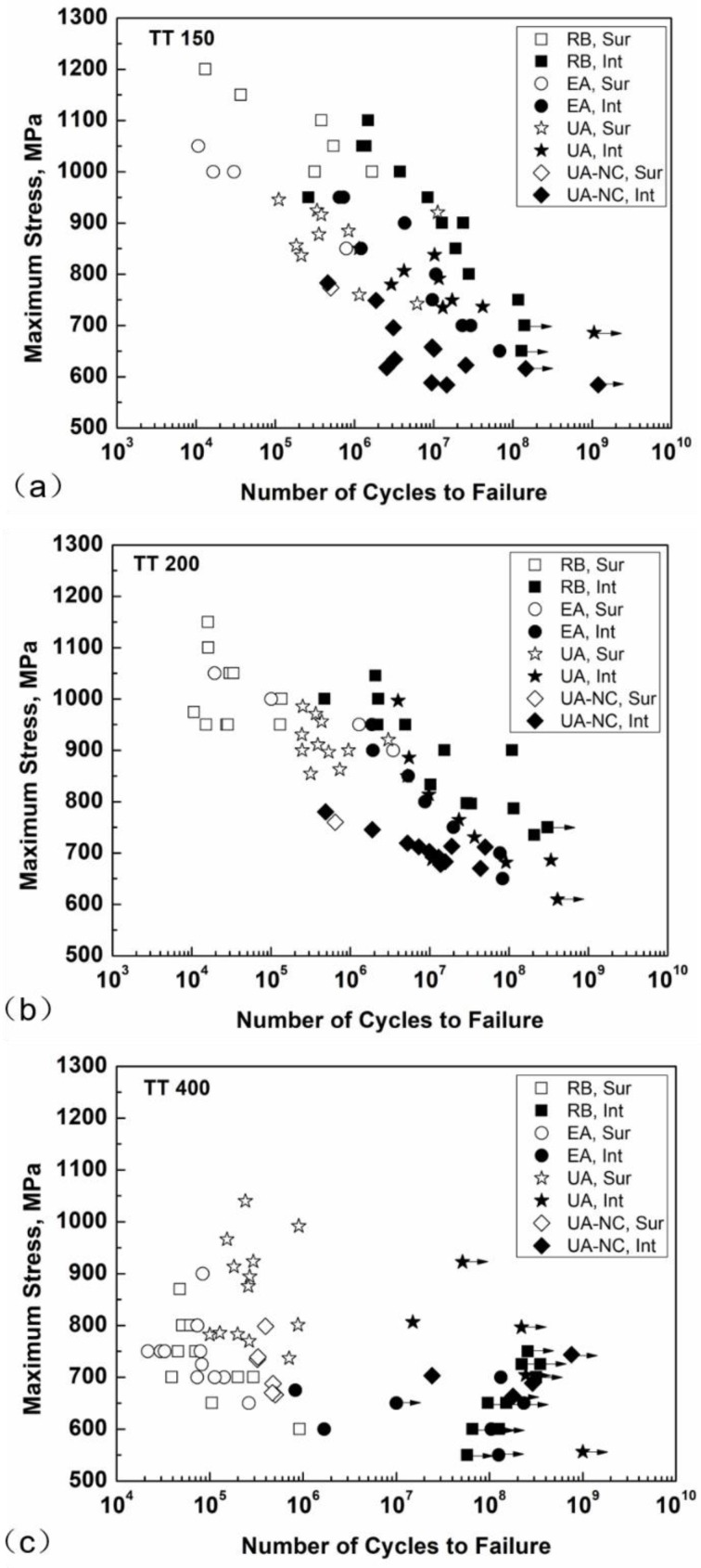
Fatigue stress–life (S-N) data for three material conditions: (**a**) TT 150; (**b**) TT 200; (**c**) TT 400. (Sur: crack surface initiation, Int: crack interior initiation, Symbol with arrow: no break).

Figure 2 shows the S-N data for the three material conditions under RB, EA, UA, and UA-NC tests. For the two high strength conditions (Figure 2a,b), the fatigue strength for the RB method is higher than that for the EA method with a difference of 50 to 200 MPa for TT 150 and 50 to 100 MPa for TT 200; the fatigue strength for the UA method is almost the same as that for the EA method in the HCF regime and is slightly higher than that for the EA method in the VHCF regime; and the data for the UA-NC method are lower than those for the other three methods. For the low strength condition (Figure 2c), the fatigue strength for the RB method is almost the same as that for the EA method; the fatigue strength for the UA method is higher than that for the UA-NC method; and the fatigue strength for the UA and UA-NC methods is higher than that for the EA method. It is also noted that the data under the UA method are of large scattering in the fatigue life range between 10^5^ and 10^6^ cycles with surface crack initiation mode. One possible reason for this is the very short testing time of 5 s for 10^5^ cycles and 50 s for 10^6^ cycles. Thus, the testing onset point and its small unstable loading period may take up a non-ignorable proportion of the total testing period, which may lead to test data scattering.

For specimens of the two high strength conditions (TT 150 and TT 200), the S-N data have a trend of duplex or continuously decreasing pattern. With the increase of fatigue life, fatigue crack initiation turns to occur from the surface of the specimen (hollow symbols in Figure 2a,b) to the interior of the specimen (solid symbols in Figure 2a,b). For specimens of the low strength condition (TT 400), fatigue cracking initiates almost from the surface of specimen (hollow symbols in Figure 2c) except for a few ones beyond 10^6^ cycles (solid symbols in Figure 2c) being internal crack initiation.

### 3.2. Fracture Surface Morphology

**Figure 3:**
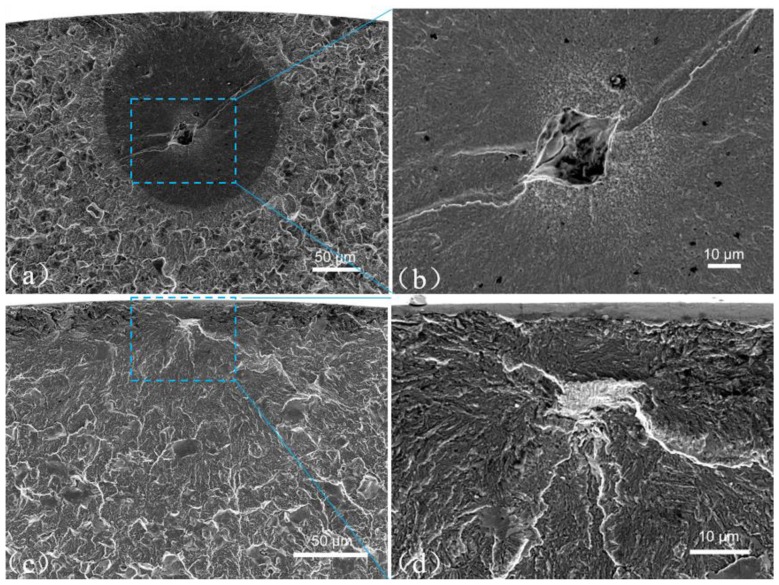
SEM photos for TT 200 by RB. (**a**) A1, *σ*_max_ = 850 MPa, *N*_f_ = 3.3 × 10^7^; (**b**) enlargement of crack origin in **a**; (**c**) A2, *σ*_max_ = 900 MPa, *N*_f_ = 1.5 × 10^7^; (**d**) enlargement of crack origin in **c**.

**Figure 4:**
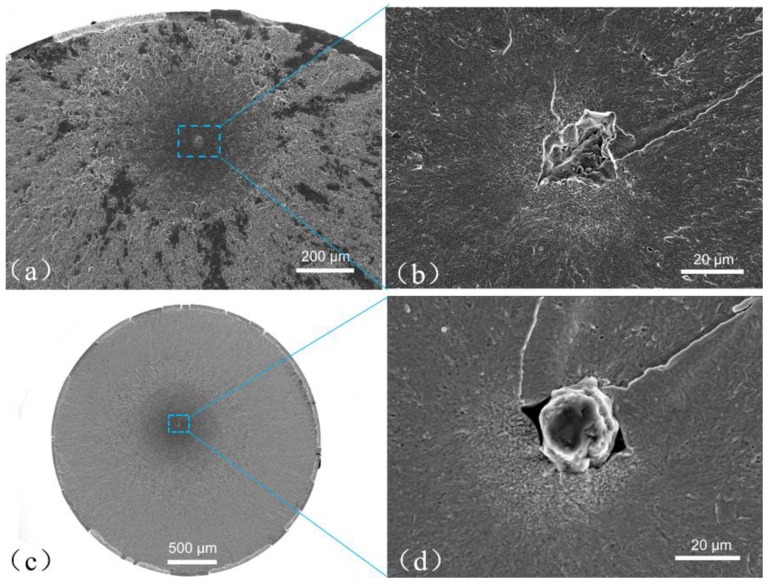
SEM photos for TT 200. (**a**) EA, B1, *σ*_max_ = 850 MPa, *N*_f_ = 5.4 × 10^6^; (**b**) enlargement of crack origin in **a**; (**c**) UA, C1, *σ*_max_ = 814 MPa, *N*_f_ = 9.8 × 10^6^; (**d**) enlargement of crack origin in **c**.

In the VHCF regime, fatigue cracking almost initiates from the interior of specimen. Figure 3 and Figure 4 show the fracture surface observations via SEM for four specimens in the VHCF regime. For RB specimens (Figure 3), crack initiation is from the subsurface of the specimen in the VHCF regime. The fracture surface of Figure 3a presents a typical FiE morphology (approximately 200 μm in diameter) being tangent to the surface of the specimen A1 (failed at 3.3 × 10^7^ cycles), and the enlargement of the crack origin of Figure 3b shows an FGA morphology surrounding an initiation site resulting from an inclusion (approximately 25 μm in diameter) as the crack origin. For interior initiation cases, fatigue cracks all initiate from an inclusion except one where the origination site is the grain boundary, which is presented in Figure 3c of the specimen A2 (failed at 1.5 × 10^7^ cycles). The high magnification of initiation region (Figure 3d) presents the intergranular feature of crack origination.

Figure 4a,b were taken of specimen B1 (failed at 5.4 × 10^6^ by the EA method), and Figure 4c,d were taken of specimen C1 (failed at 9.8 × 10^6^ by the UA method). It is seen that the images of both specimens show a clear FiE morphology containing an FGA surrounding an inclusion (with diameters of approximately 20 μm for B1 and 25 μm for C1). The location of the crack origin is rather away from the surface of the specimen compared with the case of the RB method, with the depth from surface being approximately 500 μm for B1 and 1 mm for C1.

## 4. Discussion

### 4.1. Effect of Loading Frequency

The S-N data of Figure 2 show an evident difference in fatigue strength between testing at 20 kHz (UA and UA-NC method) and at 120 Hz (EA method) for the three material conditions. Note that the strain rate of UA is 2–3 orders of magnitude higher than that of EA. The substantial increase in strain rate will lead to the intensification of atomic movement in the material so that the temperature of the specimen will rise. This phenomenon was also described as thermal dissipation induced by anelastic and inelastic deformation [45]. Hence, in the investigation of loading frequency effect, the effect of strain rate and the induced temperature rise on the strength of the material has to be identified.

**Figure 5:**
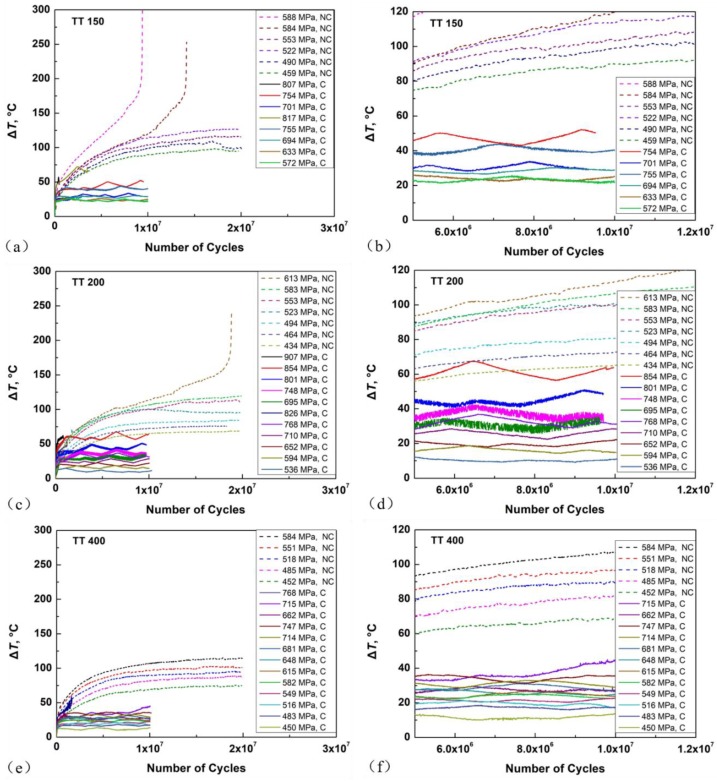
Temperature rise of UA and ultrasonic axial loading without cooling (UA-NC) specimens under different loading stresses for three material conditions. (**a**) TT 150; (**b**) enlargement of stable stage in **a**; (**c**) TT 200; (**d**) enlargement of stable stage in **c**; (**e**) TT 400; (**f**) enlargement of stable stage in **e**. (C: Cooling, NC: No Cooling).

A thermocouple system was used to monitor the temperature on the minimum cross-sectional surface of EA, UA, and UA-NC specimens. Figure 5a–f are the results of temperature measurements for UA (solid curves) and UA-NC (dashed curves) specimens under different stress levels. It is seen that temperature rise increases with loading cycles first, and then gradually becomes stabilized above 2 × 10^6^ cycles for UA specimens and 1 × 10^7^ cycles for UA-NC specimens as shown in Figure 5a,c,e. In the VHCF regime, the stable temperature rise or the generated heat is mainly caused by crack initiation as well as other inelastic deformation. When the loading stress reaches a given value, the temperature rise no longer shows stability and increases rapidly to a high value (200–300 °C), which is the damage stage due to crack propagation. In order to obtain enough data in the stable stage, we set the total acquisition period from 0 to 1 × 10^7^ cycles for UA specimens and from 0 to 2 × 10^7^ cycles for UA-NC specimens. The temperature rise by the UA method was much lower than that by the UA-NC method, suggesting that compressed air cooling effectively reduced the temperature rise of the specimens. The enlargements of the stable stage shown in Figure 5b,d,f indicate that the temperature rise increased with the loading stress for both UA and UA-NC specimens.

**Figure 6:**
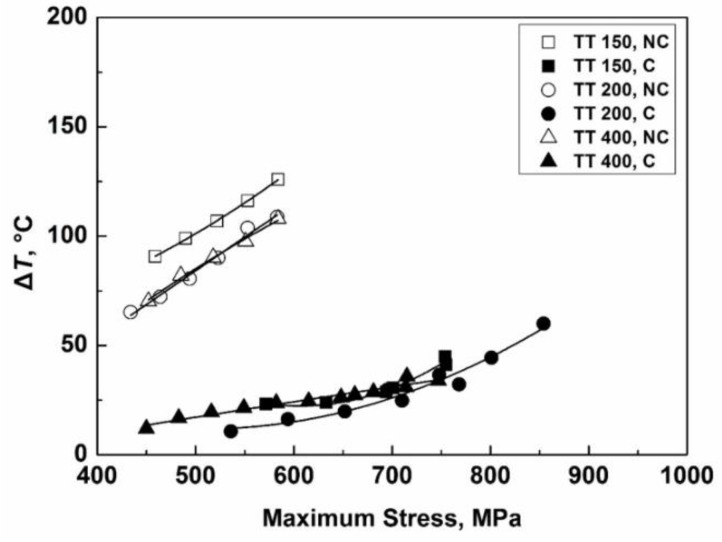
Average values of temperature rise as a function of the maximum stress by the UA and UA-NC methods for three material conditions.

Figure 6 shows the results of average temperature rise at the temperature stable stage as a function of loading stress for UA and UA-NC specimens. The average temperature rise for UA-NC specimens increases rapidly with loading stress, and the value is between 80 °C and 130 °C for TT 150, and between 70 °C and 110 °C for TT 200 and TT 400 at loading stress from 450 MPa to 600 MPa. The reason for the higher trend of temperature rise for TT 150 is probably due to the microstructure of TT 150 containing a certain amount of retained austenite [46] and the microstructure of TT 200 and TT 400 being an almost tempered microstructure [46], which may result in the slight difference in the values for thermal conductivity coefficient and specific heat capacity. For the UA method, the largest tested stress is 750 MPa for TT 150 and TT 400 and 850 MPa for TT 200, and the number of tested cycles at termination is 10^7^ (Figure 5), for which the temperature rise is in its stable stage and is in a stage of crack initiation (also refer to Figure 2). Thus, the temperature rise is mainly contributed by crack initiation. It is seen that the temperature rise is within 50 °C at stress below 800 MPa for the three material conditions for the UA method. It is obvious that the temperature rise is more noticeable for the case subjected to the UA-NC method, which implies that the frequency effect on fatigue behavior is more likely to prevail for a no-cooling situation. To the contrary, the temperature rise is evidently eased for the case subjected to the UA method thanks to the cooling action, so that the coupling effect of induced temperature with strain rate will be restricted. This also explains the values of fatigue strength under the UA-NC method being evidently lower than those under the UA method for TT 150 and TT 200 specimens, as shown in Figure 2a,b.

**Figure 7:**
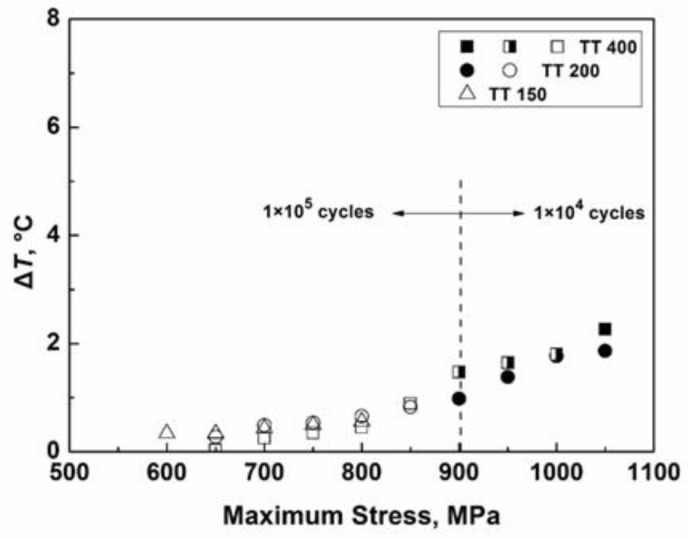
Average values of temperature rise as a function of the maximum stress by the EA method for three material conditions.

Figure 7 shows the variation of temperature rise with the maximum stress for the EA method for three material conditions. Owing to the temperature rise becoming stable rapidly, the total acquisition period was set from 0 to 1 × 10^5^ cycles under low stress, and was from 0 to 1 × 10^4^ cycles under high stress. It is seen from Figure 7 that the temperature rise also increases with loading stress, but the increment is less than 3 °C for the three material conditions, so the effect of temperature rise on the fatigue performance by the EA method is negligible.

**Figure 8:**
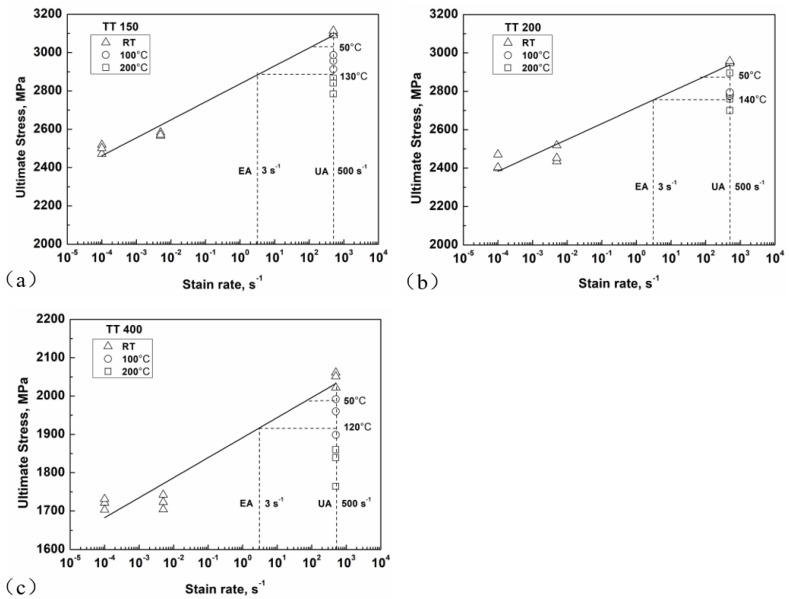
Ultimate stress under different loading strain rates and temperatures for three material conditions: (**a**) TT 150; (**b**) TT 200; (**c**) TT 400. (RT: Room temperature).

Further, monotonic mechanical tests under different strain rates (10^−4^ s^−1^, 5 × 10^−3^ s^−1^, and 500 s^−1^) at room temperature (20 °C) and under 500 s^−1^ at high temperatures (100 °C and 200 °C) were carried out by means of separated Hopkinson pressure bar testing to examine the effect of strain rate and temperature on material strength. The experimental results of Figure 8 show that the ultimate stress increases with strain rate at room temperature, and decreases with temperature at 500 s^−1^ for the three material conditions. With regard to fatigue testing under the EA and UA methods, the range of strain rate was calculated as between 2 s^−1^ and 4 s^−1^ for the EA specimen and between 300 s^−1^ and 700 s^−1^ for the UA specimen. It is obvious that the ultimate stresses of the tested material at 2–4 s^−1^ and 300–700 s^−1^ are higher than that at quasi-static condition (10^−4^ s^−1^). Therefore, it is reasonable to use the material strength at relevant strain rate and temperature in the evaluation of fatigue strength for EA and UA specimens.

In Figure 8, two vertical dashed lines are plotted to mark the corresponding strain rates of EA (3 s^−1^) and UA (500 s^−1^) specimens. In order to address the effect of temperature rise by the UA method, we defined a critical temperature *T*c (*T*c = 130 °C for TT 150, *T*c = 140 °C for TT 200, *T*c = 120 °C for TT 400). When the temperature of the UA specimen is below *T*c, the material strength of the UA specimen is higher than that of the EA specimen, and then the loading frequency effect will occur. When the temperature of the UA specimen is equal to *T*c, the material strength of the UA specimen is identical to that of the EA specimen, and then the loading frequency effect will diminish. In addition, due to the large value of temperature rise for the UA-NC specimen, it is likely that its resulting temperature is higher than *T*c; thus, the material strength and, therefore, the fatigue strength of the UA-NC specimen will be much less than those of the EA specimen.

It has been described that the temperature of the UA specimen is less than 70 °C (temperature rise 50 °C plus room temperature 20 °C) under low stress levels for the three heat treatment conditions, which is below the value of *T*c, and the material strength and the fatigue strength for the UA specimen are then higher than those for the EA specimen, i.e., the loading frequency effect may prevail. This analysis is consistent with the S-N data under low stress shown in Figure 2. 

Above all, for the three material conditions (TT 150, TT 200, and T 400), the effect of loading frequency is caused by the related strain rate under low stress. For a better understanding of the mechanism of the loading frequency effect, the Johnson–Cook formula [47] of Equation (1) is used to evaluate the effect of loading frequency on fatigue strength:

(1) $$\sigma =\left(A+B\varepsilon^{n}\right)\left(1+C\ln{\dot{\varepsilon }}^{*}\right)\left(1-T^{*}{}^{m}\right)\;$$

where ε is the equivalent plastic strain; ${\dot{\varepsilon }}^{*}=\dot{\varepsilon }/{\dot{\varepsilon }}_{0}$ is the dimensionless plastic strain rate for ${\dot{\varepsilon }}_{0}=1\;s^{-1}$; and $T^{*}=\left(T-T_{r}\right)/\left(T_{m}-T_{r}\right)$ is the homologous temperature with *T* being experimental temperature, *T_r_* room temperature, and *T_m_* melting temperature of the material. *A*, *B*, *C*, *m*, and *n* are material coefficients. The expressions in the second and the third sets of parentheses represent the effect of strain rate and temperature, respectively. Here, we define a parameter *η* as the ratio of material strength at UL frequency to CL frequency, which is expressed in Equation (2).

(2) $$\eta =\frac{\left(1+C\ln{\dot{\varepsilon }}_{\mathrm{UL}}^{*}\right)\left(1-T_{\mathrm{UL}}^{*m}\right)}{\left(1+C\ln{\dot{\varepsilon }}_{\mathrm{CL}}^{*}\right)\left(1-T_{\mathrm{CL}}^{*m}\right)}\;$$

When *η* is larger than unity, the fatigue strength at the UL frequency is higher than that at the CL frequency, which means the existence of a loading frequency effect. When *η* is equal or close to unity, the fatigue strength at the UA frequency tends to be identical with that at the CL frequency, which means that the effect of loading frequency no longer prevails.

From the testing data, we obtained the Johnson–Cook formulas for the three material conditions of TT 150, TT 200, and TT 400, which are shown in Equations (3)–(5), respectively.

(3) $$\sigma =2836.5\left(1+0.033\ln{\dot{\varepsilon }}^{*}\right)\left(1-T^{*}{}^{0.78}\right)\;$$

(4) $$\sigma =2714.6\left(1+0.030\ln{\dot{\varepsilon }}^{*}\right)\left(1-T^{*}{}^{0.839}\right)\;$$

(5) $$\sigma =1891.4\left(1+0.027\ln{\dot{\varepsilon }}^{*}\right)\left(1-T^{*}{}^{0.798}\right)\;$$

Table 2 lists the experimental data and the calculated values of *η* for UA and EA frequencies under low stress (700 MPa) in the VHCF regime. Note that the relevant temperatures of UA specimens for the three material conditions are between 46 °C and 52 °C, and the temperatures of the EA specimen are taken as 20 °C (room temperature) because of the negligible temperature rise. The related strain rates of the UA and EA specimens are 419 s^−1^ for the UA specimen and 2.5 s^−1^ for the EA specimen. The calculated values of *η* according to Equations (3)–(5) are all above unity, indicating the prevalence of the loading frequency effect for the three material conditions under corresponding low stress, which is consistent with the S-N data shown in Figure 2.

In a similar way, Table 3 lists the experimental data and the calculated values of *η* for the UA and UA-NC specimens under 584 MPa (the maximum stress in temperature measurement for UA-NC). The related stain rate is 628 s^−1^, and the values of specimen temperature are between 36 °C and 44 °C for UA specimens and between 127 °C and 146 °C for UA-NC specimens. The ratios of *σ*_UA_ to *σ*_UA-NC_ are all beyond unity, indicating that the fatigue strength of the UA specimen is higher than that of the UA-NC specimen because of the high temperature rise of the UA-NC specimen. The calculated results are consistent with the trend of S-N data shown in Figure 2.

As a consequence, it is anticipated that the methodology and the results with regard to the loading frequency effect on the HCF and VHCF performance is applicable to other cases of high-strength steels.

### 4.2. Effect of Loading Type

From Figure 2a,b, it is seen that the fatigue strength with interior crack initiation mode by the RB method is higher than that by the EA and UA methods at fatigue life above 10^6^ cycles, which is a result of different loading types: stress gradient feature on the cross section for the RB method against the stress uniform distribution on the cross section for AL, i.e., the EA and UA methods. 

**Figure 9:**
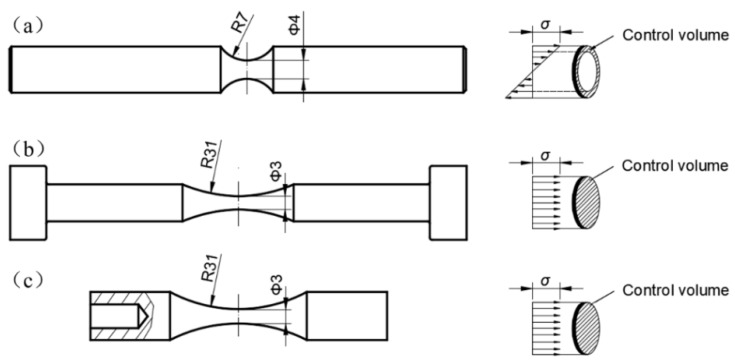
Schematic of control volume for the three types of specimens under a given loading: (**a**) RB specimen; (**b**) EA specimen; (**c**) UA specimen.

Figure 9 is a schematic of the control volume for three types of specimens under a given loading. It is seen that the control volume for the RB specimen is evidently smaller than that for the EA and UA specimens because of the presence of the stress gradient for the former. By using finite element code ANSYS, the control volume for the RB specimen (*Φ* = 4 mm, *R* = 7 mm) was calculated as 5.63 mm^3^, and that for the EA and UA specimen (*Φ* = 3 mm, *R* = 31 mm) was 32.6 mm^3^. The control volume for EA and UA specimens is almost six times that for the RB specimen. In other words, EA and UA specimens have a higher risky possibility than the RB specimen, which causes the fatigue strength by the EA and UA methods to be inferior to that by the RB method.

**Figure 10:**
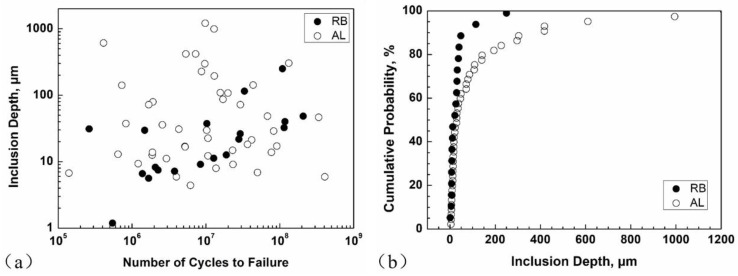
Values of inclusion depths from specimen surface versus fatigue life (**a**) and cumulative probability of inclusion depths (**b**) for RB and AL methods.

Figure 10 presents the values of inclusion depth from specimen surface and the related cumulative probability for the RB and AL methods (EA and UA). From Figure 10a, the average depths for RB and AL methods are 37 μm and 133 μm, respectively, and the range of inclusion depths under AL is apparently larger than that under RB. From Figure 10b, the cumulative probability of inclusion depths for the AL method deviates from the RB method at 50% cumulative probability and extends to a large value of inclusion depth, suggesting a random distribution of inclusion depths for the AL method. Because the control volume under AL is much larger than that under RB, fatigue cracking is likely to initiate from an inclusion at an arbitrary depth under AL especially in the VHCF regime, while it is more prone to initiate from an inclusion close to the specimen surface for the RB method. 

**Figure 11:**
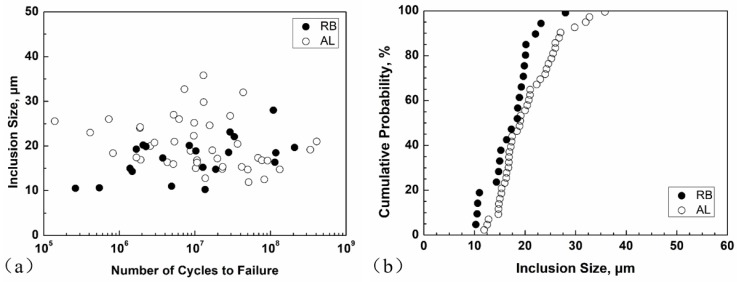
Values of inclusion sizes versus fatigue life (**a**) and cumulative probability of inclusion sizes (**b**) for RB and AL methods.

In a similar way, the size (square root of projection area) of every inclusion as crack origin was measured on fatigue-fractured surfaces and the results are shown in Figure 11. The average size of the inclusions under AL is 20 μm, which is larger than that under RB with 17 μm, and the data for the AL method are more dispersive, suggesting that the AL method with larger control volume has a high possibility of finding a larger inclusion as crack origin.

In our previous paper [43], a statistical method was proposed to evaluate the effect of specimen size on fatigue life of metallic materials in the VHCF regime by taking into account the concept of control volume, in which the fatigue performance of a large specimen was evaluated by a small specimen via the difference in the control volume for the two types of specimens. Here, the method is adopted for the assessment of the loading type effect.

Consider that the fatigue life (*N*) decreases with the increase of applied stress (*σ*) for the specimens with high strength conditions, which is expressed by Equation (6). 

(6) $$N=A\sigma^{a}\;\mathrm{or}\;\log_{10}N=a\log_{10}\sigma +\log_{10}A\;$$

Based on the data of interior crack initiation for RB specimens in Figure 2a,b, the formulas for TT 150 and TT 200 are obtained as Equations (7) and (8), respectively.

(7) $${\mathrm{TT}\;150:\;\log}_{10}N=-9.986\log_{10}\sigma +36.524\;$$

(8) $$\mathrm{TT}\;200:{\;\log}_{10}N=-13.533\log_{10}\sigma +47.062\;$$

Then, the fatigue life *N_i_* (*i* = 1, 2, …, *n*) under a stress level *σ_i_* is transformed to an arbitrary given stress $\sigma^{\prime }$; that is $N_{i}^{\prime }$ as in Equation (9):

(9) $$N_{i}^{\prime }=\frac{{\sigma^{\prime }}^{a}}{\sigma_{i}{}^{a}}N_{i}\;\mathrm{or}\;\log_{10}N_{i}^{\prime }=a\log_{10}\frac{\sigma^{\prime }}{\sigma_{i}}+\log_{10}N_{i}\;(i=1,\;2,\;\cdots ,\;n).$$

If $\sigma^{\prime }$ is taken as 700 MPa, a group of $N_{i}^{\prime }$ can be calculated, which (in logarithm to base 10) can be fitted by the Weibull distribution as in Equation (10):

(10) $$F(x)=\{\begin{array}{cc} 1-e^{-{\left(x/\lambda \right)}^{k}} & x\geq 0\\ 0 & x<0 \end{array}\;$$

where λ is the scale parameter and *k* is the shape parameter. The values of λ and *k* were then obtained from the fitted cumulative distribution of $N_{i}^{\prime }$ in logarithm to base 10. As previously mentioned, the control volume of the EA specimen (*V*_EA_ = 32.6 mm^3^) is about six times as large as that of the RB specimen (*V*_RB_ = 5.63 mm^3^), and we can deduce the cumulative distribution function of fatigue life *N*^EA^ of the EA specimen with *n* = 6 by Equation (11).

(11) $$\begin{array}{l} F_{N}^{\mathrm{EA}}(x)=P\left\{N^{\mathrm{EA}}\leq x\right\}=P\left\{\min\left\{N_{1}^{\mathrm{RB}},N_{2}^{\mathrm{RB}},\cdots ,N_{n}^{\mathrm{RB}}\right\}\leq x\right\}\\ =1-P\left\{N_{1}^{\mathrm{RB}}>x\right\}P\left\{N_{2}^{\mathrm{RB}}>x\right\}\cdots P\left\{N_{n}^{\mathrm{RB}}>x\right\}=1-{\left[1-F(x)\right]}^{n}\\ =\{\begin{array}{cc} 1-e^{-n\;{\left(x/\lambda \right)}^{k}} & x\geq 0\\ 0 & x<0 \end{array} \end{array}$$

Thus, we were able to get the fatigue life under 700 MPa at the reliabilities of 50%, 90%, 95%, 99%, and 99.9% for the EA specimen. In a similar way, the fatigue life under other stress levels of 750 MPa, 800 MPa, 850 MPa, 900 MPa, 950 MPa, 1000 MPa, and 1050 MPa at the concerned reliabilities for the EA specimen were obtained successively. Finally, the derived P-S-N (probability-stress-life) curves for the EA specimen were readily plotted. 

**Figure 12:**
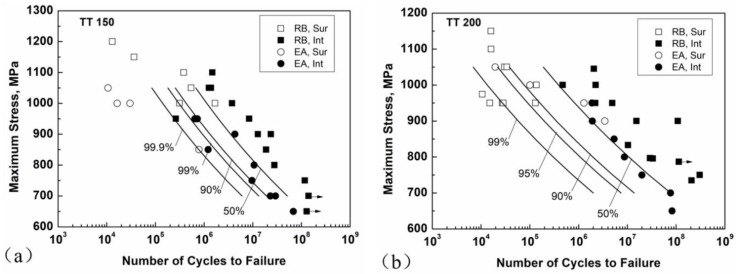
P-S-N (probability-stress-life) curves for the EA specimen (*V*_EA_ = 32.6 mm^3^) derived from experimental data of the RB specimen (*V*_RB_ = 5.63 mm^3^) for TT 150 (**a**); and TT 200 (**b**) together with tested S-N data.

The derived P-S-N curves together with tested S-N data for TT 150 and TT 200 are shown in Figure 12. The predicted P-S-N curves of the EA specimen (*V*_EA_ = 32.6 mm^3^) were derived from RB data (*V*_RB_ = 5.63 mm^3^) with interior crack initiation mode, which were generally consistent with the experimental data for the EA method. That is to say, the effect of loading type is basically caused by the difference in control volume. This statistical method is a suitable approach to reconcile the data under different loading types with different values of control volume.

It is noticed that the above results were based on the interior crack initiation data for specimens TT 150 and TT 200. The fatigue strength for specimens TT 400 by RB and EA methods (Figure 2c) does not show a significant difference. The reason for this is that most of those failed specimens resulted from surface crack initiation, which was slightly affected by the difference of control volume.

## 5. Conclusions

In this paper, the effects of loading frequency and loading type on fatigue performance for a high-strength steel with three heat treatment conditions were studied. The conclusions are drawn as follows:

(1) For the specimens of the two high strength conditions, the fatigue strength with interior crack initiation mode for the RB method is superior to that for the EA and UA methods because of the small control volume for the former; the fatigue strength for the UA method is slightly higher than that for the EA method in the VHCF regime, suggesting the existence of a loading frequency effect; and the fatigue strength of the UA-NC method is substantially lower than that of the EA and UA methods due to the temperature rise of the specimen for the former.
(2) For the specimens in the low strength condition, the fatigue strength for the RB method is almost the same as that for the EA method because fatigue cracking almost initiates from the specimen surface; thus, the loading type effect is diminishing. The fatigue strength for the UA method is higher than that for the UA-NC method, and the fatigue strength for both the UA and UA-NC methods is higher than that for the EA method, showing an evident loading frequency effect.
(3) The combined response of strain rate and the induced temperature rise is the reason for the loading frequency effect. A parameter *η* was proposed to judge whether the loading frequency effect may occur, and the calculated results are in agreement with the experimental data.
(4) The statistical method used based on the control volume is suitable for reconciling the effect of loading type. The predicted P-S-N curves of the EA specimen are consistent with the experimental data for the specimens of the two high strength conditions.

## Figures and Tables

**Table 1:** Mechanical properties for three heat treatment conditions.

Heat Treatment Condition	Tensile Strength, MPa	Yield Strength, MPa	Microhardness, Hv
TT 150	2497	NA	828
TT 200	2425	1764	754
TT 400	1718	1583	526

**Table 2:** Values of experimental parameters for UA and EA specimens and results of *η*.

Specimen Condition	*σ*, MPa		$\dot{\varepsilon },\;s^{-1}$		*T*, °C		*η*
	UA	EA	UA	EA	UA	EA	
TT 150	700	700	419	2.5	51.7	20	1.103
TT 200	700	700	419	2.5	46.0	20	1.108
TT 400	700	700	419	2.5	51.1	20	1.080

**Table 3:** Values of experimental data for UA and UA-NC specimens and results of *σ*_UA_/*σ*_UA-NC_.

Specimen Condition	*σ*, MPa	$\dot{\varepsilon },\;s^{-1}$	*T*, °C		*σ*_UA_/*σ*_UA-NC_
			UA	UA-NC	
TT 150	584	628	43.1	145.9	1.134
TT 200	584	628	36.3	128.7	1.107
TT 400	584	628	43.7	127.9	1.106
